# Nanomedicine Particles Associated With Chemical Exchange Saturation Transfer Contrast Agents in Biomedical Applications

**DOI:** 10.3389/fchem.2020.00326

**Published:** 2020-04-22

**Authors:** Yanlong Jia, Kuan Geng, Yan Cheng, Yan Li, Yuanfeng Chen, Renhua Wu

**Affiliations:** ^1^Department of Radiology, Second Affiliated Hospital, Shantou University Medical College, Shantou, China; ^2^Department of Radiology, The First People's Hospital of Honghe Prefecture, Mengzi, China

**Keywords:** chemical exchange saturation transfer (CEST), drug delivery systems, magnetic resonance imaging, nanomedicine, nanoparticles, theranostic

## Abstract

Theranostic agents are particles containing both diagnostic and medicinal agents in a single platform. Theranostic approaches often employ nanomedicine because loading both imaging probes and medicinal drugs onto nanomedicine particles is relatively straightforward, which can simultaneously provide diagnostic and medicinal capabilities within a single agent. Such systems have recently been described as nanotheranostic. Currently, nanotheranostic particles incorporating medicinal drugs are being widely explored with multiple imaging methods, including computed tomography, positron emission tomography, single-photon emission computed tomography, magnetic resonance imaging, and fluorescence imaging. However, most of these particles are metal-based multifunctional nanotheranostic agents, which pose potential toxicity or radiation risks. Hence, alternative non-metallic and biocompatible nanotheranostic agents are urgently needed. Recently, nanotheranostic agents that combine medicinal drugs and chemical exchange saturated transfer (CEST) contrast agents have shown good promise because CEST imaging technology can utilize the frequency-selective radiofrequency pulse from exchangeable protons to indirectly image without requiring metals or radioactive agents. In this review, we mainly describe the fundamental principles of CEST imaging, features of nanomedicine particles, potential applications of nanotheranostic agents, and the opportunities and challenges associated with clinical transformations.

## Introduction

Cancer remains one of the most threatening diseases to human health. Currently, the standard therapeutic regimens for cancer include surgery, radiation therapy, and adjuvant chemotherapy (Allhenn et al., 2012). Among these, adjuvant chemotherapy forms a significant part of medicinal strategies, even in cases that are considered unresectable (Li et al., 2016). However, the clinical outcome of chemotherapeutic drugs is discouraging due to their severe side effects, multidrug resistance, and insufficient drug delivery to the tumor areas (Chan et al., 2014a). Creating a platform that can overcome the disadvantages of chemotherapeutic drugs and improve their tumor curative effects is therefore essential.

Nanoparticles (NPs), with their advantages of high surface-area-to-volume ratio, high drug payload capacity, high sensitivity, multimodal signaling capacity, unique size, and fewer adverse effects, are an ideal platform for improving the medicinal effect of drugs on cancer treatment (Pan et al., 2009; Jokerst and Gambhir, 2011; Mao et al., 2016). Additionally, encapsulating medicinal drugs and imaging contrast agents (CAs) into a single platform can enable providing real-time feedback on the pharmacokinetics, monitoring location, and biodistribution of the target site, which can help to predict treatment responses (Lammers et al., 2011). NPs have shown remarkable success as both medicinal and diagnostic agents, thereby showing potential as a single platform capable of combining active or passive drug delivery targeting, environmentally receptive drug release, molecular imaging, and other medicinal functions (Yue et al., 2017). Such systems have collectively been described as nanotheranostics, which is an emerging field that uses nanoscale materials to collect diagnostic information for well-informed cancer therapy, which is especially vital for establishing personalized treatment routines that improve outcomes and reduce side effects (Janib et al., 2010). To develop effective multifunctional nanotheranostic agents, the imaging sensitivity, target accuracy, and drug release control need to be considered. Presently, nanotheranostic particles incorporating medicinal drugs are being extensively explored with multiple imaging methods, including computed tomography (CT), positron emission tomography (PET), single-photon emission computed tomography (SPECT), magnetic resonance imaging (MRI), and fluorescence imaging (Janib et al., 2010). However, each imaging method and theranostic agent have advantages and disadvantages. For example, metal-based (namely, Gd, Fe, or Mn) nanotheranostic agents possess multifunctional features but also increase the potential risks of toxicity. MRI provides limited physiological or biochemical information (Kato and Artemov, 2009; Choi et al., 2011), whereas PET and SPECT can provide early-stage tumor metabolic information but require the injection of high-cost radioactive agents, thus limiting their broad application (Medricka et al., 2019). Furthermore, the intrinsic depth limitation of fluorescence imaging hinders its broad application, despite its high sensitivity (Lian et al., 2019). Hence, alternative non-metallic and biocompatible nanotheranostic agents are urgently needed.

Recently, nanotheranostic agents combining medicinal drugs and chemical exchange saturated transfer (CEST) contrast agents have shown good promise. CEST is a new MRI approach based on the theory of magnetization transfer (MT) and chemical exchange that utilizes a selective radiofrequency (RF) irradiation pulse on exchangeable protons, thus resulting in a loss of the bulk water proton signal intensity (Mao et al., 2016). When detected, the bulk water signal change indirectly reveals information about solute protons. Since Ward and Balaban first proposed it in 2000 (Ward et al., 2000), the CEST approach has gained popularity, because it enables the amplified detection of low-concentration molecules and can be turned “on” and “off” when required by changing the frequency-selective RF irradiation pulses. It can also concurrently identify multiple agents with distinct exchangeable protons because each resonates with a specific frequency (Winter, 2012). Notably, CEST MRI has the potential to supply information on metabolites in biological tissues as well as anatomical features. Furthermore, CEST contrast agents can be precisely adapted to react to a given stimulus (e.g., pH, enzymes, temperature, and metabolite levels) (Langereis et al., 2009; Liu et al., 2012; Daryaei et al., 2017; Zhang et al., 2017; Sinharay et al., 2018), which provides benefits for imaging sensitivity and specificity. Most importantly, CEST contrast agents represent an attractive alternative to metals or radioactive agents, and they show unaltered pharmacokinetic and safety profiles, which are essential for nanotheranostic agent development.

In this review, we mainly described CEST imaging fundamental principles, features of nanomedicine particles, potential application of nanotheranostic agents, and clinical opportunities and challenges.

## Basic Principles OF CEST Imaging

**Figure 1:**
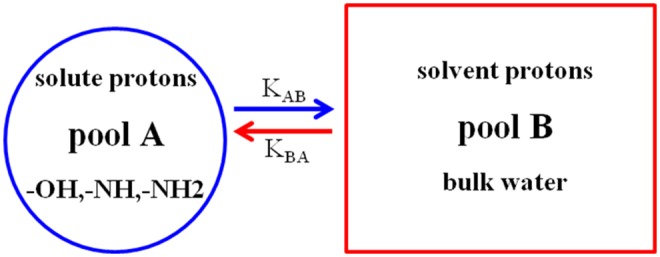
The basic principle of CEST experiment (dual pool model). Pool A (solute) is in exchange with pool B (solvent).

The basic principles of CEST imaging are principally based on the phenomenon of MT and chemical exchange between exchangeable protons and bulk water protons (Dou et al., 2019). A dual pool model is often used to analyze the CEST imaging mechanism (Van Zijl and Yadav, 2011). Typically, pool A represents a solute containing exchangeable protons with a specific frequency offset, and pool B represents a solvent that provides bulk water protons (Figure 1). When a frequency-selective RF saturated pulse is applied to pool A, the saturated solute protons in pool A transfer to the bulk water protons of pool B via the chemical exchange process, resulting in the loss of the bulk water signal, which in turn enhances the MRI contrast. Notable, when the solvent concentration in pool B is about 110 M (molar), which is much higher than the pool A solute concentration (in the micromolar or millimolar range), a single saturation transfer would be inadequate to show any perceptible effect on the bulk water protons. However, if the solute protons have a suitably fast exchange rate and lengthy saturation time, this process continually repeats, thus serving as an amplification and eventually becoming evident on the water signal (Mao et al., 2016). That is why CEST imaging enables the indirect amplification of detectable solutes at low concentration. Additionally, this mechanism of CEST allows turning the CEST signal “on” and “off” by simply changing the RF saturation pulse parameters.

Naturally, not all solute protons provide the CEST effect, which requires certain conditions. When pool A is saturated by an RF pulse, a positive exchange rate (K_AB_) and a reverse exchange rate (K_BA_) exist in the two pools. Typically, for CEST to occur efficiently, the following condition must be met: **K**_AB_ < Δ**ω**_AB_, where Δ**ω**_AB_ is the chemical shift differential between the exchangeable pool A and pool **B** (expressed by Δ**ω**_AB_ = **ω**_A_ - **ω**_B_) (Castelli et al., 2013; Mcmahon and Bulte, 2018). Another condition is K_AB_ ≥ R_1A_, that is, the positive exchange rate must be faster than the longitudinal relaxation rate (R_1A_) of the exchangeable protons. In short, K_AB_ ≥ R_1A_ achieves effective saturation, and **K**_AB_ < Δ**ω**_AB_ ensures better resolution (Zhou et al., 2004; Zhou and Van Zijl, 2006). Earlier studies showed that the CEST mechanism is affected by many factors but primarily by the (i) exchange rate; (ii) number of exchangeable protons or concentration; (iii) saturation time and RF pulse power; (iv) pH and temperature; and (v) field strength (Castelli et al., 2013; Daryaei et al., 2017; Mao et al., 2019). Among these factors, the exchange rate most involved in determining the CEST efficiency. A higher exchange rate increases the CEST signal sensitivity. However, if the exchange rate of solute protons is too high or too low, then no CEST signal will appear (Soesbe et al., 2013). When the exchange rate is too high (**K**_AB_ >Δ**ω**_AB_), the K_BA_ is increased, and the resolution is insufficient to display the CEST signal. If the exchange rate is too low (**K**_AB_ < Δ**ω**_AB_), fewer saturation exchangeable protons transfer to the bulk water protons in a given time, and thus, the CEST signal is too weak for observation. Zhou et al. (2004) reported that as the main magnetic field strength (B_0_) increases, both Δ**ω** and the exchange rate increase, while R_1A_ decreases. Enhancing the magnetic field strength may be an effective way to increase the sensitivity of the CEST. Unfortunately, the higher field strength not only dramatically increases the MT effect but also increases the chance of reaching the specific absorption rate (SAR) safety limitation (Simegn et al., 2019). Hence, the effectiveness of a CEST agent may be improved by maximizing both the exchange rate and frequency shift (Δ**ω**).

According to the MT ratio asymmetry (MTR_asym_), the CEST contrast scale can be quantified using the following formula (Wang et al., 2019):

$$\begin{array}{l} \text{MTRasym}=\left(\text{S}-\Delta \omega -\text{ S}+\Delta \omega \right)/{\text{S}}_{0} \end{array}$$

Where S –Δ**ω** and S +Δ**ω** are the signal intensities obtained by saturation at the negative and positive sides of the CEST spectrum, also called the Z-spectrum, respectively. S_0_ is the signal intensity of bulk water without saturation. The Z-spectrum is often used to provide insights into the interpretation of the exchange mechanism and the physics phenomenon. In the Z-spectrum, 0 ppm represents the bulk water maximum saturation point. Typically, most values are offset from water, and those marked as having a positive frequency are situated on the left side of the Z-spectrum, while negative frequencies are situated on the right side. As mentioned above, the uniformity of the B_0_ magnetic field is important for CEST imaging. An inhomogeneous B_0_ field shifts the Z-spectrum relative to the zero frequency of water, thereby weakening the CEST effect. Therefore, using the water saturation shift reference (WASSR) approach is essential for the shimming correction before scans (Kim et al., 2009).

## Features of Nanomedicine Particles

Image-guided drug delivery is essential for personalized medicinal routines. Several studies have shown that some anticancer drugs have CEST effects, while others do not. For example, Li et al. reported that more than 22 anticancer drugs have CEST effects (Li et al., 2016). However, anticancer drugs directly injected into the body for CEST imaging and treatment were limited by the high dose requirements, highly toxic side effects on normal tissues, and the possible CEST signal reduction by physiological or pathological factors. Therefore, a single platform that can combine both CEST contrast agents and medicinal drugs is urgently required. NPs may be an ideal platform owing to several unique features: (i) Their high surface-area-to-volume ratio, which enables the incorporation of various functionalities to reduce adverse effects on the surface, minimal disruption of the drug itself, prolonged drug circulation times, enhanced tumor targeting, and improvement of the medicinal efficacy of drugs. (ii) Their unique small size tends to be selective toward tumor sites, where they rapidly accumulate. This is known as the enhanced permeability and retention (EPR) effect (including leaky vasculature and decreased lymphatic drainage) and provides a higher signal-to-noise ratio in tumors. Further, they offer a (iii) high drug payload capacity and large numbers of exchange protons conjugated on the surface, which allow detecting the amplified CEST signal even at low dosages of contrast agent, and (iv) high biocompatibility and biodegradability without toxic byproducts (Wu et al., 2010; Jokerst and Gambhir, 2011; Mao et al., 2016; Reessing and Szymanski, 2019). In short, NPs offer a promising platform not only to further enhance CEST imaging sensitivity but also to improve the medicinal effect of the drug, while having various other biomedical applications.

## Potential Applications of Nanotheranostic Agents

**Figure 2:**
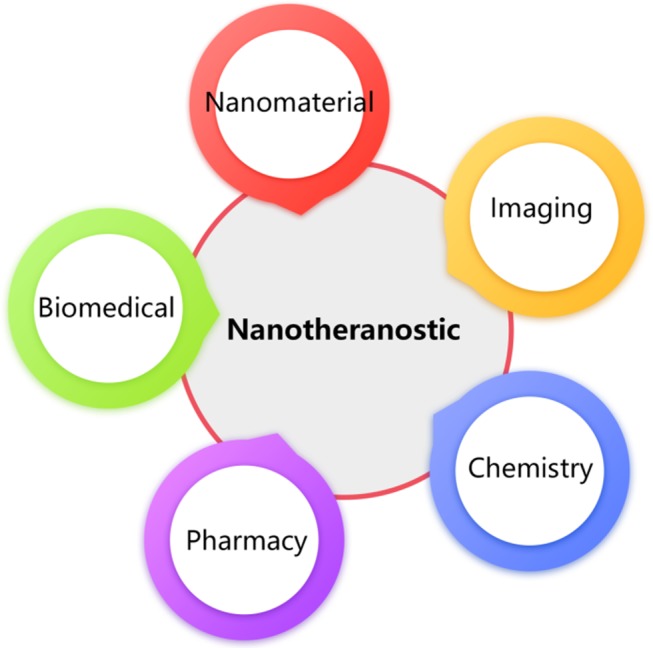
Schematic represent that nanotheranostic was a highly interdisciplinary field, including the biomedical, imaging, chemistry, pharmaceutical sciences, and nanotechnology.

Nanotheranostic agents offer advantages by combining disease diagnosis and therapy within a single nanoparticle formulation to provide real-time information on the pharmacokinetics, as well as the location and distribution of target sites, but challenges are inevitable. Because nanotheranostics is highly interdisciplinary, involving fields including biomedicine, imaging, chemistry, pharmaceutical sciences, and nanotechnology, its development has been relatively slow compared to that of single diagnostic or medicinal agents (Figure 2) (Lammers et al., 2011). Nonetheless, metal-based nanotheranostic agents have been successfully used in pre-clinical and clinical research (Lacerda and Toth, 2017). For example, drug-loaded superparamagnetic NPs and Gd- or Mn-conjugated nanomedicine enable monitoring not only drug delivery but also drug release by observing the change in T_1_ and T_2_/${\text{T}}_{2}^{*}$ relaxation times after the drugs were released (Choi et al., 2011; Liu et al., 2019; Ruan et al., 2019). Although metal-based nanotheranostic agents have shown promise for cancer therapy, metal deposition, and potential toxicity risks hinder their future development in clinical use. Furthermore, indirectly detectable MRI signals reflect only the drug carriers rather than the drug itself, and it remains unknown whether the pharmacokinetic profiles of the contrast agents and the liberated drugs correspond well. Therefore, alternative novel contrast methods based on the natural properties of tissues without employing metal or other lanthanides are urgently needed. Non-metal-based CEST MRI contrast agents are promising for monitoring the drug delivery process. Consequently, drug delivery systems, including liposomes, metal, mesoporous silica, and virus NPs, dendrimers or dendritic polymers, micelles, and scaffolds are being co-loaded with medicinal and CEST agents for pre-clinical and clinical research (Table 1).

**Table 1:** Nanotheranostic agents with the diaCEST effects in preclinical applications.

**Nanotheranostic**	**Parameters (*in vivo*)**	**Results**	**Chemical shift**	**Models**	**References**
BA/DOX-liposome	11.7 T, TR = 5.0 s, TE = 21. 6 ms, B_1_ = 4.7 μT T_sat_ = 3 s	Liposomes+ TNF-α group (1.5%) was higher than liposomes (0.4%) group	5.0 ppm	CT26 tumor	(Chan et al., 2014b)
BA/MPP-liposome	11.7 T, TR = 5.0 s, TE = 21. 6 ms, B_1_ = 4.7 μT T_sat_ = 3 s	BA/MPP-liposome group was ~4% (peak at post- 90 min), and liposomal MPP group was ~1% (peak at post- 30 min)	5.0 ppm	Vaginal mucus	(Yu et al., 2015)
Citicoline-liposome	11.7 T, TR = 5.0 s, TE = 6 ms, B_1_ = 2.7 μT Tsat = 3 s, total time = 5 min	Citicoline-liposomal delivery could be directly monitored and quantified via CEST MRI	2.0 ppm	Ischemic stroke	(Liu et al., 2016)
PEG-PAM-PAN@Dox	7.0 T, TR = 6.0 s, TE = 27.6 ms, B_1_ = 1.5 μT Tsat = 5 s, total time = 13 min 13 s	Nanomedicine both have the CEST effect and therapy; MTR _asym_ was ~2.17 vs. ~0.09% (post-injection vs. pre-)	0.5 ppm	Breast cancer	(Jia et al., 2019)
Olsa-RVRR NPs	11.7 T, TR = 5 s, TE = 3.7 ms, B_1_ = 3.6 μT, Tsat = 4 s, total time = 9 min 20 s	Furin-mediated intracellular self-assembly; Olsa-NPs have a good antic-cancer effect and CEST was pH independence	9.8 ppm	HCT116 LoVo	(Yuan et al., 2019)
SA-Dendrimer	11.7 T, TR = 5.5 s, TE = 4.8 ms, B_1_ = 3.4 μT, T_sat_ = 2.2 s, time = 6 min 19 s	SA-dendrimers present a promising new nanoplatform for medical applications	9.4 ppm	U87 glioma	(Lesniak et al., 2016)
Dextran-polymers	11.7 T, TR = 5s, TE = 5 ms, B1 = 1.8 μT, T_sat_ = 3 s, total time = 20.7 min	MTR _asym_ of dextran+ TNF-α group was higher than dex150 alone (~4.2 vs. ~0%)	1.0 ppm	CT26 tumor	(Chen et al., 2019)
Pem-hydrogels	11.7 T, TR = 5 s, TE = 6 ms, B1 = 3.6 μT, T_sat_ = 3 s	Pem-peptide hydrogels have the potential of monitoring drug distribution and release	5.2 ppm	GL261	(Lock et al., 2017)
PS-PPF scaffold	11.7 T, TR = 6 s, TE = 16.5 ms, B_1_ = 4.7 μT, T_sat_ = 4 s	PS-coated 3D-PPF have the potential to monitor drug delivery and drug release	1.8 ppm 3.6 ppm	PC12 cells	(Choi et al., 2011)

*BA, Barbituric acid; MPP, mucus-penetrating particles; PEG-b-P(AM-co-AN), polyethylene glycol-polyacrylamide-polyacetonitrile; Dox, doxorubicin; NPs, Nanoparticles; Olsa, Olsalazine; SA, Salicylic acid; Pem, pemetrexed; PS, protamine sulfate; PPF, poly(propylene fumarate); IONP, iron oxide nanoparticles; MONP, manganese oxide nanoparticles*.

### Liposomes

Liposomes are the most versatile particles for delivering nanotheranostic agents, and they have been successfully embedded with medicinal drugs and CEST contrast agents for preclinical and clinical studies. For example, Chan et al. (2014b) loaded the diamagnetic CEST (diaCEST) contrast agent barbituric acid (BA) and the medicinal drug doxorubicin (Dox) into liposomes, which were intravenously injected into the tail of mice to target and treat CT26 colon tumors. They successfully observed an increased nanotheranostic agent accumulation within the tumor areas by CEST MRI (Chan et al., 2014b). Their results showed that the co-treatment with tumor necrosis factor-alpha (TNF-α) had higher treatment potency based on synergistic effects (with an average CEST contrast of 1.5%) than co-treatment without TNF-α (with an average CEST contrast of 0.4%). BA was very well-suited to be a diaCEST contrast agent due to its large frequency offset from water (peak at 5.0 ppm), high loading into liposomes, and good biocompatibility. In addition, these liposomes were developed based on the formulation of the clinically used DOXIL® and therefore hold potential for clinical translation. Thus, they have implications for future clinical trials on liposomes and other NPs. Recently, Yu et al. designed liposomal-based mucus-penetrating particles (MPPs) loaded with BA to monitor their vaginal distribution and retention by CEST imaging (Yu et al., 2015). Their results demonstrated that the liposomal-based MPPs co-loaded with medicinal drugs could overcome the mucus barrier and provided a uniform distribution and sustained delivery of mucosal drug, thus exhibiting immense potential to improve the monitoring and treatment of diseases. Liu et al. also packaged citicoline in liposomes as a theranostic agent, and citicoline maintained its inherent CEST effect (2 ppm) to image-guide the treatment of ischemic stroke tissue (Liu et al., 2016). Citicoline is a natural supplement used to treat neurodegenerative diseases and possesses good neuroprotective properties. Their results showed that the delivery of liposomal citicoline to the ischemic stroke tissue could be monitored in real time and quantified using CEST MRI without the need for chemical labeling or any extra imaging agents. This not only facilitates rapid clinical translation for citicoline but also provides a useful method for monitoring the effectiveness of citicoline delivery in individual patients to achieve personalized medicine.

As previously mentioned, although liposome-based paramagnetic (PARA) CEST agents have great potential for molecular imaging and drug delivery, their clinical application is limited owing to the potential toxicity of metallic ions. Pan et al. recently developed lipid-encapsulated perfluorocarbon (PFC) NPs carrying PARACEST contrast agents and medicinal drugs, making them an extremely useful platform for molecular imaging and drug delivery and release (Pan et al., 2009). Lipid-encapsulated PFC-based nanoemulsions have high sensitivity and specificity, thus avoiding interference from the surrounding background. The combined application of PFC and the diaCEST contrast agent conjugate as a promising theranostic agent currently being further explored.

### Nanoparticles

**Figure 3:**
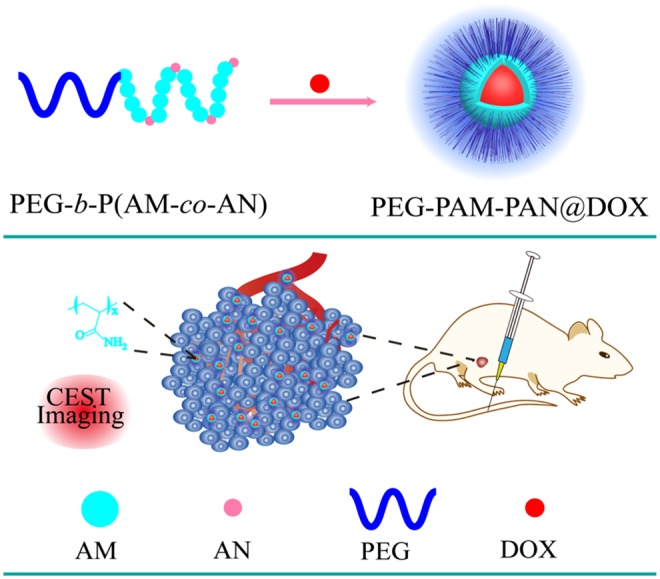
Schematic diagram of the fabrication of PEG-PAM-PAN@DOX for chemotherapy and CEST imaging. Reproduced from Jia et al. (2019) under a Creative Commons Attribution (CC BY) license.

NPs are promising as nanocarriers that avoid the drawbacks of traditional chemotherapy drugs and enhance their curative effects using image guidance. For example, our research team successfully synthesized a NP [polyethylene glycol-polyacrylamide-polyacetonitrile, PEG-*b*-P (AM-co-AN)] with an inherent CEST MRI signal at 0.5 ppm for treating breast cancer (Jia et al., 2019). The results showed that the nanomedicine loaded with Dox (PEG-PAM-PAN@DOX) could not only treat breast cancer but also enabled monitoring its accumulation in the tumor areas using CEST MRI (Figure 3). Additionally, the surface of the PEG-PAM-PAN@DOX prolonged drug exposure time, which is important for improving medicinal indices. However, the frequency offset of PEG-PAM-PAN@DOX was too small and easily confused with the surrounding background. Moreover, the NP had no target and was limited by the drug-load capacity. Recently, Yuan et al. demonstrated that the furin-mediated intracellular self-assembly of Olsalazine (Olsa) NPs enhanced both the CEST MRI signal and the tumor therapy efficacy (Yuan et al., 2019). They conjugated the anticancer drug Olsa with the cell-penetrating peptide RVRR to create a single small-molecule probe Olsa-RVRR for CEST imaging with a large frequency shift (at 9.8 ppm) relative to that of water. Subsequently, Olsa-RVRR enters intracellularly during the furin-mediated self-assembly of Olsa-NPs, which then inhibit the DNA methylation for tumor therapy. *In vitro* and *in vivo* findings show that Olsa-NPs provide a good anti-cancer effect, long drug circulation time, and a pH-independent OlsaCEST signal. The advantage of pH independence would enable applying Olsa in different furin-expressing tumor models without considering the interference of endogenous tumor-derived pH changes. Finally, it is our hope that this study will inspire further attempts to effectively design enzyme-responsive agents that could be also used as a theranostic platform for CEST imaging and cancer therapy.

### Dendrimers or Dendritic Polymers

Dendritic polymers serve as classical compounds with immense biomedical application potential because of their specific properties (including low polydispersity and rigidity), and their surfaces can be easily modified, making them particularly well-suited for CEST imaging and medicinal agents (Gonawala and Ali, 2017; Mcmahon and Bulte, 2018). Ali et al. reported the feasibility of using dendritic PARACEST agents to simultaneously track the pharmacokinetics of two differently sized NPs *in vivo* (Ali et al., 2009). Snoussi et al. observed that polyuridylic acid [poly(rU)], as single-stranded RNA, could be used for both treatment and CEST imaging (frequency offset from water at 6 ppm) (Snoussi et al., 2003). If it associates with cationic dendrimers that could easily cross the anionic barrier of cell membranes, it could provide insights into a detection model for gene therapy and would open a novel avenue for future research that may contribute to designing an ideal *in vivo* gene delivery vehicle. Salicylic acid (SA), a metabolite of aspirin, serves as an anti-inflammatory drug that is widely used in clinics. Most importantly, SA derivatives possess large chemical shifts from water (peak at 9.4 ppm) and can be conjugated to poly(amidoamine) (PAMAM) dendrimers to form a theranostic agent. Lesniak et al. successfully developed SA conjugated to PAMAM dendrimers as a versatile platform for tunable, high performance CEST imaging to monitor its distribution in mice carrying U87 glioblastoma (Lesniak et al., 2016). In the future, SA-based PAMAM dendrimers co-loaded with medicinal drugs that target the specific proteins of brain tumors [e.g., integrins and epidermal growth factor receptor (EGFR)], have the potential to improve drug retention within tumors and to augment the medicinal effects.

**Figure 4:**
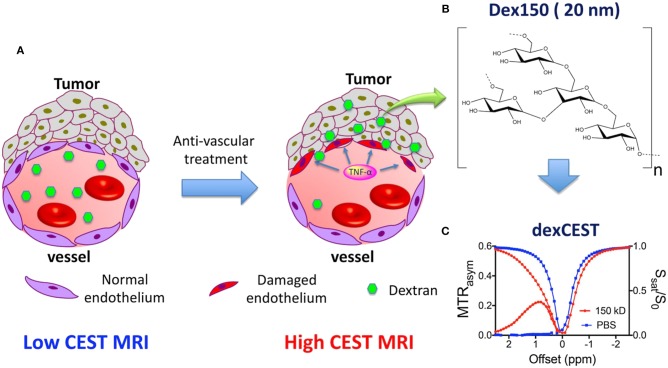
Illustration of using dexCEST MRI to monitor the tumor responses to antivascular therapies. **(A)** Schematic of the effect of antivascular therapies such as TNF-α on the extravasation of dextran molecules. The picture on the left shows a “normal” vessel around tumor cells, consisting of endothelial cells but lacking pericyte coverage. While it has greater permeability than healthy vessels, molecules larger than the pore size cannot easily pass. Upon TNF-α treatment, as shown in the picture on right, tumor endothelial cells are selectively damaged by TNF-α, resulting in an enormous augmentation of vessel permeability and strong extravasation of large molecules in the tumor. **(B)** Chemical structure of Dex150. **(C)** CEST characteristics of Dex150 in PBS as shown by the Z spectrum and MTRasym plot of 3.6 mg/mL mM Dex150 (20 mM per glucose unit or 24 μM per dextran molecule, in 10 mM PBS, pH = 7.3). CEST MRI was performed using a 4-s-long CW radiofrequency pulse (B 1 = 3.6 μT) at 37°C. Reproduced from Chen et al. (2019) with permission from Copyright 2019 John Wiley & Sons.

Recently, sugar-based polymers have been investigated for CEST MRI detection and theranostic systems (Li et al., 2018; Han and Liu, 2019). Dextran has a well-proven safety profile and is widely used in clinics without the risk of accumulation and toxicity, even at very high doses. Chen et al. demonstrated that dextran, with an inherent CEST signal at 1.0 ppm, can be used to observe tumor permeability changes in response to a vascular-disrupting agent (Figure 4) (Chen et al., 2019). By simply monitoring the changes in the dextran CEST signal in the tumor at various time intervals, the beginning of vascular disruption can be determined. The future of nanotheranostic research using dextran-based polymers conjugated with medicinal drugs is very promising. Future research should aim to optimize the time frame for vascular-disrupting agents, which can enhance the tumor uptake of chemotherapeutic agents.

### Micelles

**Figure 5:**
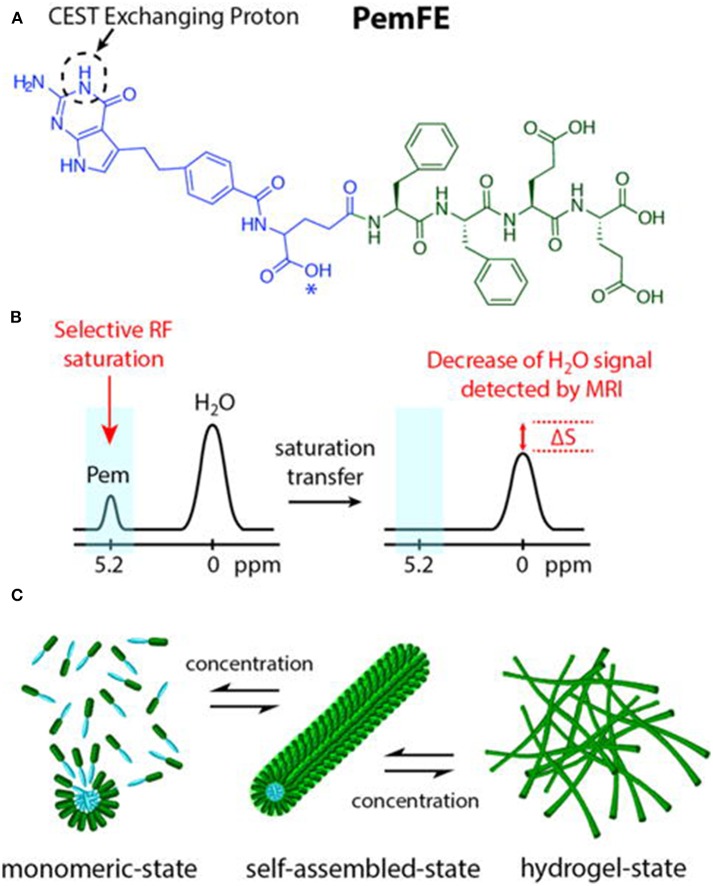
**(A)** Chemical structure of the studied PemFE molecule with the CEST MRI signal originating from the aromatic amine exchangeable proton on Pem (blue). A possible PemFE structural isomer conjugation site is indicated by an asterisk (^*^). **(B)** CEST contrast is measured by a decrease in water signal when the selectively saturated 5.2 ppm exchangeable proton is within supramolecular filaments and hydrogels. **(C)** Schematic illustration of the self-assembly of PemFE monomers into filamentous nanostructures that can further entangle into a 3D network for formation of self-supporting hydrogels under suitable conditions (pH, concentration, and ionic strength). Reprinted with permission from Lock et al. (2017). Copyright (2017) American Chemical Society.

Micellar formations are an attractive structure for diagnostic imaging and drug delivery owing to their uniform size, high stability, ease of formation, and good biocompatibility and biodegradation. Further, they enable integrating various functionalities into a single structure. Lock et al. reported a supramolecular strategy to convert pemetrexed (Pem)-peptide to a molecular hydrogelator with an inherent CEST effect at 5.2 ppm for nanotheranostic use in a mouse glioma model (Figure 5) (Lock et al., 2017). The concept of self-assembling drug-peptide conjugates permits precisely controlling a high drug load at the molecular design level. Uniform distribution in the tumor was observed by CEST MRI 2 h post-injection. CEST imaging 4 days post-injection still detected the Pem-peptide hydrogel and indicated a larger, more uniform distribution. All these results demonstrated Pem-peptide nanofiber hydrogels enabled the *in vivo*, non-invasive monitoring of the drug release, drug location, and distribution. This whole hydrogel approach only comprises the designed drug conjugate and water, thus affording a single label-free theranostic platform.

Tissue engineering is a novel biomedical technology offering great advantages for tissue replacement and restoration. Furthermore, it has the potential for loading gene therapy agents into the target cells because of its good biocompatibility and biodegradability. Most importantly, engineered tissues, such as hyaluronic acid (HA) hydrogels and poly(amido amines) (PAA) polyamide materials, contain more mobile exchange protons and can potentially be applied *in vivo* for CEST imaging. Therefore, drug delivery and tissue regeneration based on engineered tissues show great promise in both imaging guidance and disease treatment. HA, as a major component of extracellular matrices, has been used for drug delivery, tissue regeneration, and CEST imaging (Varghese et al., 2009; Shazeeb et al., 2018). Recently, Dou et al. successfully crafted novel HA hydrogels and PAA polymers with unique CEST signals that enabled guidance during the hydrogel optimization process for regenerative medicine, drug delivery applications, or both (Dou et al., 2019). Although the research on CEST properties in engineered tissues is still in its initial stages, the first *in vivo* evidence obtained is essential for further developing and optimizing these biomaterials and achieving clinical translation.

### Scaffold

Using biodegradable 3D poly(propylene fumarate) (PPF) scaffolds as the matrix for drug release is relatively new, but incorporating water-soluble drugs into PPF is challenging because of its hydrophobicity. Nevertheless, Choi et al. successfully used porous PPF scaffolds to load Dox-coated iron oxide nanoparticles (IONPs) and manganese oxide nanoparticles (MONPs) as vehicles for prolonged anti-cancer drug release (Choi et al., 2011). The observed changes in the T_1_ and T_2_ values caused by Dox-coated IONPs and MONPs indicated the drug release. Interestingly, protamine sulfate (PS), as a small cationic protein with inherent CEST signals at 1.8 ppm (guanidine proton) and 3.6 ppm (amide proton), can be coated on the 3D-PPF scaffold surface to directly monitor the bio-polymer release (e.g., proteins, polypeptides) using CEST MRI without the need for intermediate metallic compounds. These findings indicate a novel system that can monitor drug release, growth factors, and cytokines, which is particularly important for tailoring personalized treatments. Additionally, PS has two relatively large labile groups that cause a chemical shift, which make PS very suitable for use in the ratiometric CEST approach, as it reduces the compounding effects derived from the concentration of the contrast agent. PS thus has good prospects for wide application (Chen et al., 2017).

### Virus Nanoparticles

The architecture of virus NPs contains interior and exterior areas, which are easily modified, either chemically or genetically, to attach differentiated specific targeting groups. The natural cell-targeting capability of virus NPs enables carrying CEST MRI contrast agents and chemotherapeutic drugs to diseased tissues by altering the virus biology (Wu et al., 2010). For example, Vasalatiy et al. successfully designed viral-based PARACEST agents for molecular targeted imaging (Vasalatiy et al., 2008). As viral-based CEST NPs develop in the future, they are expected to potentially conjugate medicinal genes or targeted drugs for gene- or cell-level therapy. They could thus become an interesting platform for nanotheranostic agents.

### Mesoporous Silica Nanoparticles

Mesoporous silica nanoparticles (MSNs), which have uniform mesopores, good biocompatibility, and large surface areas, can serve as a nanocarrier for ion complexes, and MSNPs have been extensively used in preclinical applications. Ferrauto et al. demonstrated that PARACEST agents anchored on the surface of MSNs had a higher sensitivity than those anchored on dendrimers or micelles (Ferrauto et al., 2014). However, these findings were demonstrated only in *in vitro* studies. Thus far, *in vivo* applications have been constrained by the complex synthesis of the PARACEST agents and the potential toxic effects of metal. Undeniably, however, the outer surface and inner pores of the silica framework afford large spaces that can potentially attach specific drug payloads and imaging probes. Thus, MSNs could finally fulfill the multimodal functions of drug release and imaging diagnosis in the future.

### Drugs With CEST Effects

**Figure 6:**
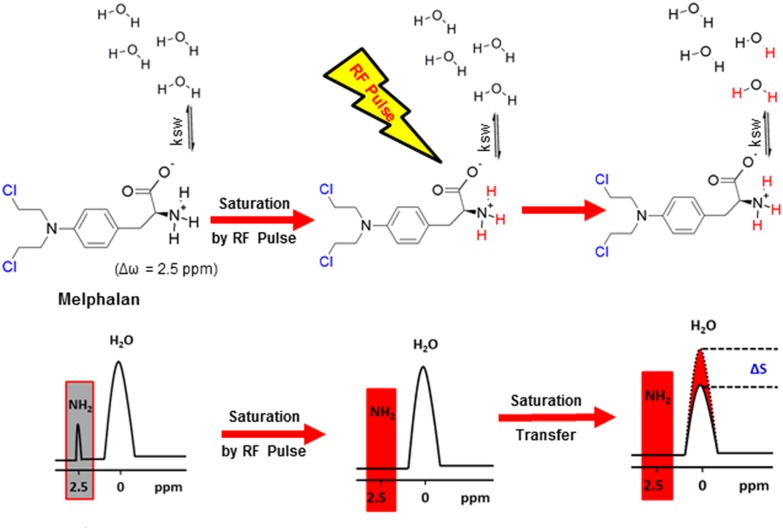
Mechanism of CEST MRI contrast generation by melphalan. Saturated melphalan amine protons are highlighted in red. The biologically active site of melphalan is highlighted in blue (the Cl -leaves for DNA alkylation by the activated carbocation to occur). Ksw is the exchange rate constant of the labile melphalan protons with exchangeable protons of endogenous biological molecules represented by bulk water protons. Reprinted with permission from Ngen et al. (2016). Copyright (2016) American Chemical Society.

As mentioned above, drugs have inherent CEST effects. For example, Ngen et al. demonstrated that the DNA alkylating anti-cancer agent (melphalan) could be directly monitored by CEST MRI to observe its *in vitro* medicinal response without the need to conjugate or modify the drug (Ngen et al., 2016). This is extremely important because even a small modification can significantly reduce drug activity or change its target. Their results demonstrated that melphalan had an inherent CEST signal at 2.5 ppm, which was pH-dependent and peaked at a pH of 6.2 (Figure 6). Upon cell death, the cellular pH decreased from 7.4 to 6.4, thus amplifying the melphalan CEST signal, making melphalan very suitable for the CEST MRI monitoring of its medicinal response. Additionally, 24 h after treatment with melphalan, the CEST signal peak at 3.3 ppm broadened. It was speculated that this broadening was caused by melphalan entering the cells and interacting with the DNA to form melphalan-DNA adducts. However, direct *in vivo* application of melphalan is still limited and needs further study.

## Opportunities and Challenges

### Opportunities

Feasibly, nanotheranostic agents containing both CEST imaging contrast agents and medicinal drugs on a single platform can enable early disease detection and improving the treatment efficacy. They can provide the ability to rapidly assess and modify treatments to address the requirements of individual patients, while also prompting the further development nanotheranostic agents. Concurrently, many nanocarriers (e.g., liposomes and micelles) have been approved for clinical trials, and CEST MRI has been successfully transferred to clinical applications (Mao et al., 2016; Lin et al., 2018). All these findings indicate that nanotheranostic agents are a very promising way to improve people's health. In addition, in excess of 45 nanoparticulate drug formulations have gained clinical approval, and Phase I–III clinical trials are currently being conducted on at least 200 products (Davis et al., 2008). Tailoring some of these formulations into nanotheranostic systems using the CEST MRI approach will enable directly using this approach in clinical practice to prioritize patients and facilitate personalized medicine.

### Challenges

Although nanotheranostic agents show promise in both CEST imaging and therapy, they still present various challenges: (i) Metal-based nanotheranostic agents are toxic, are easily recognized by the reticuloendothelial system (RES), and are rapidly cleared out. (ii) The dosage and circulation time for *in vivo* application must be reasonably weighted because imaging diagnosis has a short duration and a low-dosage design, while medicinal applications require a longer circulation duration and higher dosage. (iii) The pharmacokinetic profiles of the imaging agents and the liberated drugs currently do not match. (iv) Theoretically, nanotheranostic agents are biodegradable, but the potential hazards of long-duration body retention are unknown. (v) Finally, the detailed interpretation of the CEST signal and its clinical application may be affected by many factors, such as B_1_ and B_0_ inhomogeneities, long scan duration, and high power deposition, thus highlighting the need for a unified standard (Van Zijl and Yadav, 2011; Vinogradov et al., 2013; Ferrauto et al., 2016). Hence, these issues must be addressed to further develop nanotheranostic agents and to realize their clinical transformation.

## Conclusions

Nanotheranostic agents combining the complementary advantages of CEST MRI and NPs have great potential for transformation into clinical applications. Although the exact nanotheranostic mechanisms are still not completely understood, and although various challenges must still be resolved, improving the MRI hardware, image acquisition schemes, and nanotechnology may lead to a promising future for nanotheranostics. Undeniably, future advancements require multidisciplinary cooperation in the fields of chemistry, nanotechnology, pharmaceutical sciences, and biomedical imaging. Medicinal drugs using real-time imaging-guided visualization play an important role in individualized treatment and appear to be a promising approach to completely understanding the disease components, processes, dynamics, and therapies at the molecular level. In summary, in the near future, interest in possible applications of this method is very likely to increase, and its further development will expand its applicability.

## Author Contributions

YJ and RW conceived and designed the project. YJ contributed to the writing and editing of the final review. KG, YCheng, YL, and YChen conducted the literature research. Renhua Wu critically revised the manuscript. All the authors approved the final draft.

## Conflict of Interest

The authors declare that the research was conducted in the absence of any commercial or financial relationships that could be construed as a potential conflict of interest.
